# Renal cell carcinoma with intramyocardial metastases

**DOI:** 10.1186/1471-2490-14-73

**Published:** 2014-09-06

**Authors:** Anna M Czarnecka, Pawel Sobczuk, Fei Lian, Cezary Szczylik

**Affiliations:** 1 Department of Oncology, Military Institute of Medicine, Warsaw, Poland; 2 The Second Faculty of Medicine with the English Division and the Physiotherapy Division, Medical University of Warsaw, Warsaw, Poland; 3 Emory University School of Medicine, Atlanta, GA, USA; 4 Department of Oncology, Military Institute of Medicine, Laboratory of Molecular Oncology, Szaserow 128, 04-141 Warsaw, Poland

**Keywords:** Renal cell carcinoma, Myocardium, Metastasis, Pazopanib, Axitinib

## Abstract

**Background:**

Cardiac metastases from renal cell carcinoma without vena caval involvement are extremely rare with a limited number of cases reported in the worldwide literature until now. Nevertheless, this rare location of metastasis may significantly influence patient treatment and prognosis. Cooperation between oncology, cardiology, and urology teams are indispensable in cases of patients suffering from intramyocardial tumors. For these individuals, treatment guidelines based on large-scale studies are unavailable and only case/case series analysis may provide clinicians with decision assistance.

**Case presentation:**

In this paper, we report a case of a 50-year-old Caucasian male diagnosed with a 10.2 × 10.3 × 10.0 cm lower pole left renal mass in January 2002. He was subsequently treated with immunochemotherapy, tyrosine kinase inhibitors (TKIs), and mTOR inhibitors (mTORIs) - that is sunitinib, everolimus, and sorafenib. In March 2012, contrast-enhancing tumors in the left myocardium (∅22 mm) and in the interventricular septum (∅26 mm) were seen on CT. Cardiology testing was conducted and the patient was treated with pazopanib with a profound response. Overall survival since the clear cell renal cell carcinoma (ccRCC) diagnosis was 11 years 2 months and since diagnosis of multiple heart metastases was 1 year.

**Conclusions:**

Cardiac metastases present a unique disease course in renal cell carcinoma. Cardiac metastases may remain asymptomatic, as in the case of this patient at the time of diagnosis. The most common cardiac presentation of renal cell carcinoma is hypertension, but other cardiac presentations include shortness of breath, cough, and arrhythmias. Targeted systemic therapy with tyrosine kinase inhibitors may be useful for this group of patients, but necrosis in the myocardium can result in tamponade and death. Regular cardiac magnetic resonance imaging scans are required for treatment monitoring.

## Background

Rare locations of metastasis may significantly influence patient treatment and prognosis. For these individuals, treatment guidelines based on large-scale studies are unavailable, and only case/case series analysis may provide clinicians with decision assistance. The incidence of primary and secondary heart tumors in unselected pathology necropsy reports is about 0.17% with secondary tumors more common. Secondary cardiac tumors are often found incidentally in even up to 20% of patients with metastatic cancer, sarcoma, or lymphoma [1]. Metastases spread to the pericardium, myocardium, and endocardium in descending order of frequency [2].

In general, cardiac metastases are 20 to 40 times more common than primary cardiac malignancies, and have been reported in different studies in approximately 2%to 18% of cases at autopsies. Intramyocardial metastases arise most often in the course of malignant melanoma, leukemia, lymphoma, and lung, esophageal and breast cancers [3,4]. At the same time, heart involvement *via* the inferior vena cava (IVC) is a well-known phenomenon in clear cell renal cell carcinoma (ccRCC) cases. Renal cell carcinoma (RCC) is known for invading the renal vein and further promoting tumor thrombosis of the vena cava and even the right atrium [1]. For these patients, long-term outcome after radical surgical treatment with RCC and tumor thrombus extension reaching up to the right atrium justifies an extensive procedure with median survival (including in-hospital mortality) of 25 months. Cardiopulmonary bypass with deep hypothermic circulatory arrest allows safe and precise extirpation of all intracaval and intracardiac tumor mass [5]. Manual repositioning of the tumor thrombus out of the right atrium into the inferior vena cava on the beating heart is also a safe and feasible approach with low risk of tumor thrombembolization [6].

In the absence of IVC involvement, cardiac metastases are exceptional in ccRCC with a limited number of cases reported in the worldwide literature (Table 1) [3]. No cases of well-documented cardiology diagnostics or oncological follow-ups with noted progression-free survival (PFS) and overall survival (OS) have been described in the literature before (Tables 1 and 2). No such cases have been reported in clinical trial of recently widely used sunitinib, sorafenib, pazopanib or axitinib [7-10]. In this report, we present the first case of a patient with intramyocardial metastases treated with tyrosine kinase inhibitors (TKI), who was carefully monitored.

**Table 1:** Blood test results on diagnosis and treatment of presented case

**Blood test**	**16.04.**	**01.08.**	**02.08.**	**08.01.**	**30.01.**	**15.02.**
	**2012**	**2012**	**2012**	**2013**	**2013**	**2013**
**CK**	47	49	58	20	16	16
***(N = 55-70) [U/l]***						
**CKMB**	39	11	13	13	11	23
**(N = 0–16) [U/l]**						
**Troponin I**	0.070	0.055	0.066	0.097	0.076	0.076
***(N <0.035, MI > 0.12) *** **[ng/ml]**						
**NT-proBNP**	799.4	-	-	-	1875.0	1879.3
**N < 125 [pg/ml]**						
**LDH**	243	-	178	-	134	196
**(135–232) [U/l]**						
**Treatment**	Pazopanib	IFN	IFN	Axitinib	BSC	BSC

**Table 2:** Summary of all reported cardiac intramyocardial metastases in clear cell renal cancer and the course of disease in those patients

***Case no***	***Localization***	***Reference***	***Years from nephrectomy***	***Signs or symptoms***	***Diagnostic method***	***Treatment; treatment efficacy***
**1**	LV	[11]	23	Weight loss	CT, TTE, MRI, CA	ND
**2**	LV	[12]	18	Dyspnea	CT, CA	Surgery - successful, 6 years follow-up
**3**	LV	[13]	7	Chest pain	TTE, TEE, CT, B	Chemotherapy - no response
**4**	LV	[4]	0	ND	PET-CT	ND
**5**	LV	[14]	ND	Dyspnea	CT, TTE	ND
**6**	LV, PE	[3]	8/12	Dyspnea, asthenia, and inferior limb edema, peripheral cyanosis	TTE	No
**7**	LV, PE	[15]	ND	ND	ND	Surgery - successful
**8**	RA	[16]	ND	Asymptomatic	TTE, CT	Surgery - successful
**9**	RA, LA, LV, PE	[13]	7	Endocarditis	TTE, CT, CA	Chemotherapy - no response
**10**	RV	[17]	19	ND	ND	Surgery - successful
**11**	RV	[18]	18	Asymptomatic	PET-CT	Sunitinib, everolimus - successful PR 6 months
**12**	RV	[1]	4.5	Arrhythmia, tachycardia	MRI, EBCT, CA, ECG, TEECG	Immunotherapy - no response
**13**	RV	[19]	5	Congestive heart failure (NYHA class III)	MRI, CT, CA, TEE	Echo-guided coil embolization - successful, 19 months follow-up
**14**	RV	[20]	0	Pansystolic murmur	TTE, MRI	ND
**15**	RV	[21]	0	Syncope, T wave abnormality, prolonged QT interval	ECG, TTE	Surgery - successful
**16**	RV	[22]	ND	Presyncope	TTE, B	ND
**17**	RV	[23]	ND	Asymptomatic	ND	ND
**18**	RV	[24]	ND	ND	X-ray	Surgery – died
**19**	RV	[25]	ND	Dyspnea	Post-mortem diagnosis	ND
**20**	RV, SE	[26]	4	cardiac murmur, monomorphic ventricular tachycardia	TTE	Sunitinib, ICD
**21**	SE	[2]	20	Raynaud’s-like phenomena, systolic ejection murmur	TTE, MRI, TEE, B	Surgery -successful

Legend: TTE - Transthoracic echocardiography; TEE – transesophageal echocardiography, PA – pulmonary arteries, RA – right atrium, RV – right ventricle, SE- septum, CA - Coronary angiography, B- biopsy; ICD - implantable cardioverter-defibrillator, ND – no data available; EBCT - electron-beam CT; TEECG – trans -esophageal ECG; S-S; M-M; PE - pericardial effusion.

## Case presentation

We report a case of a 50-year-old Caucasian male who was diagnosed with a 10.2 × 10.3 × 10.0 cm lower pole left renal mass in January 2002. Subsequently, he underwent radical left nephrectomy in that same month. Pathology of the resected tumor was ccRCC, Fuhrman Grade 2. In the following year, metastasis in the right adrenal gland was diagnosed and the patient was referred for a metastasectomy, which was performed in September 2003, when a 6.1 × 5.5 × 4.9 cm metastatic tumor was removed. Subsequently, he was enrolled in immunochemotherapy treatment.

Between September 2003 and January 2004, the patient received two courses of immunochemotherapy with a regimen of vinblastine, interleukin-2, and interferon alpha-2a (IFN-α-2a) in a single dosage of 4 mg intravenous vinblastine per m^2^ of body surface, 4.5 MU/m^2^ subcutaneous interleukin-2 q12h, qw1,3,5 and 3 MU/m^2^ subcutaneous interferon alpha-2a qd, qw 2,4,6 [27]. The immunochemotherapy was terminated due to stenocardial chest pain without myocardial ischemia signs. After metastasectomy and immunochemotherapy, the patient remained disease free until 2006 when multiple right lung and right femoral bone metastases were diagnosed at a routine check-up. In September 2006, bi-lobectomy of the right lung was performed, and in November 2006, the patient underwent a total right hip replacement. Total rib replacement with subsequent rehabilitation enabled the patient to walk without walking aid (cane, crutches or walker) until final deterioration (see below). All surgical specimens were confirmed as ccRCC.

In March 2007, new lung lesions were detected, and in April 2007 the patient was started on immunotherapy of 9 MU subcutaneous interferon alpha-2a qd, qw 1,3,5 with moderate tolerance of body temperature elevation up to 100.0°F, grade 3 neutropenia, myalgia, arthralgia and bone pain. The lung disease progressed after seven months from the beginning of the immunotherapy and he was transferred to sunitinib treatment beginning in November 2007 at a standard dose and schedule (50 mg, 4/2 weeks). After six cycles, the dose was reduced to 37.5 mg due to hand-foot syndrome and mucositis grade 3. The patient continued sunitinib treatment with the best response of stable disease (SD) and received a total of 15 cycles. Pamindronate disodium 90 mg administered monthly was prescribed in parallel due to bone lesions with concurrent hypercalcemia. In October 2009, the patient underwent a femoral bone intramedullary locking ChM nail placement in order to prevent pathologic fracture.

Due to further disease progression in the skeletal system, the patient was rescheduled on everolimus 10 mg/day. This treatment was continued with the best response of SD for the next 23 months (2009–2011), and was accompanied by zoledronic acid injections. As a result of a solitary new metastasis in the right kidney, the patient was referred for nephron-sparing surgery (NSS), which was performed in August 2010. In April 2011, multiple new skin metastases developed and the patient was referred for surgical consultation. In May 2011, scalp metastases were removed. After four weeks’ recovery in July 2011, the patient started sorafenib (2 × 200 mg b.i.d.) treatment. From 2011 to2012, sorafenib treatment was continued with SD for 7 cycles on standard dose. The patient tolerated the treatment well with reported neuropathies grade 2. Standard follow-up contrast-enhanced-CT scan was performed after 9 sorafenib cycles (March 2012). Metastases in the thoracic and abdominal lymph nodes, liver, right kidney, pancreas and skeleton were stable, but new lesions in the right lung (33 × 17 mm), in the right lumbar retroperitoneal region (27 × 20 mm), and in the psoas major muscle (∅10 mm) were described.

**Figure 1:**
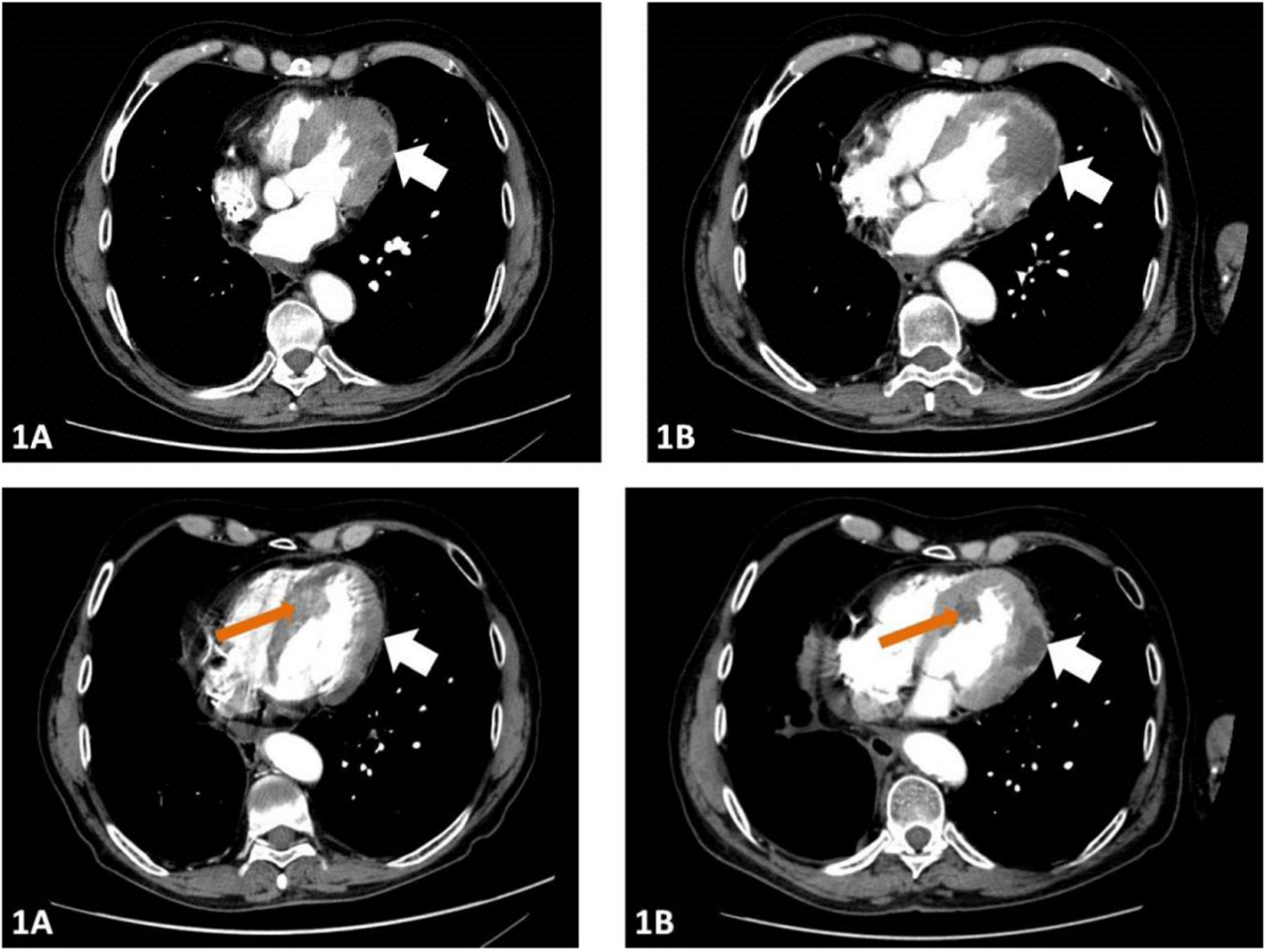
First CTimaging showing intramyocardial tumor in the LV in the left myocardium (white arrow 1A and 1B) and in the interventricular septum (red arrow 1A and 1B).

**Figure 2:**
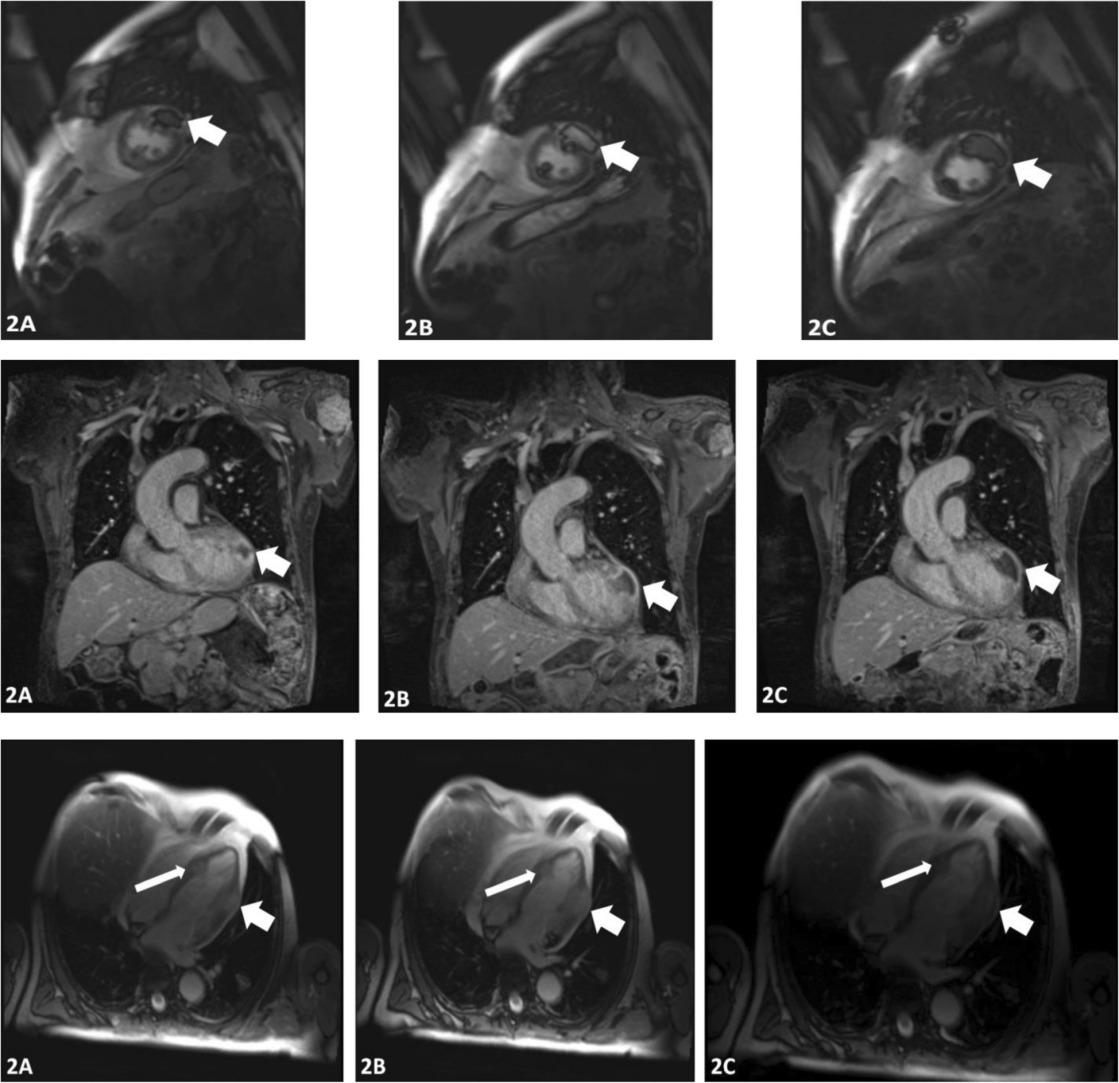
Cardiac-MRI performed on treatment onset and follow-up showing multiple (2A, 2B, and 2C) intramyocardial tumors and large necrosis on TKI treatment - coronal, horizontal and sagittal sections.

Moreover, contrast-enhancing tumors in the left myocardium (∅22 mm) and in the interventricular septum (∅26 mm) were described (Figure 1). This was defined as disease progression and the patient was transferred to pazopanib treatment in April 2012. In parallel, further laboratory tests and medical imaging were carried out (Table 2, Figure 2). In order to specify the diagnosis, we referred the patient for an echocardiogram that confirmed a 16 × 19 mm tumor localized between the inferoseptal and apical septal segments with an estimated ejection fraction (EF) of 60%.A subsequent cardiac MRI performed in April 2012 revealed multiple intramyocardial metastases (Figure 2), including tumors localized in segments: 1) apical inferior and apical lateral (∅13 × 7 mm); 2) apical septal and mid-inferoseptal (3 tumors: ∅10 mm, 11 mm, and 14 mm); 3) basal anterior (∅13 mm); 4) basal anteroseptal (∅18 mm); 5) mid-anteroseptal with pericardium infiltration (44 × 31 × 26 mm); and also in 6) anterior (∅14 mm) and posterior papillary (∅13 mm) muscles of the left ventricle (LV). On cardiac MRI, the EF was measured at 49.6% and hypokinetic muscles were described in the septum and anterolateral mid-ventricular segment. Cardiac single-photon emission computed tomography (SPECT) confirmed impaired myocardial perfusion in the inferolateral wall of the LV and anterior apex. Although the patient developed no signs or symptoms of cardiac dysrhythmia, a 24-hr Holter electrocardiogram (ECG) was performed and revealed ST-segment elevation, atrial extrasystoles, premature ventricular complexes, and a single episode of atrial fibrillation.After five months of standard pazopanib treatment, a cardiac MRI in July 2012 (Figure 2) revealed SD with massive necrosis in the tumor localized in septal segments and an EF of 45%. At this point, the 24-h Holter ECG revealed no rhythm abnormalities. Due to massive necrosis – a typical TKI treatment effect – the patient was referred for IFN-alpha treatment and received IFN-α-2a 3 million U, three times weekly for two months. This treatment was terminated due to poor tolerance with significant myalgia, arthralgia, and severe bone pain. In August 2012, a follow-up CT revealed progression according to RECIST-1.1 criteria with new lung and peritoneal tumors. In November 2012, axitinib treatment in a reduced dose (5 mg) was initiated. After two cycles of treatment, the patient’s condition deteriorated and the patient passed in March 2013. OS since ccRCC diagnosis was 11 years 2 months, since first TKI treatment was 5 years 3 months, and since diagnosis of multiple heart metastases was 1 year.

## Conclusions

Metastatic ccRCC involving the heart is well-recognized but a rare phenomenon, mostly arising as an intravascular protruding tumor. According to literature since 2000, right atrial in-growth of RCC was recently diagnosed in no more than 1% of cases at the time of nephrectomy. In post-mortem studies, cardiac metastasis was shown to be present in 11% of patients who died of RCC [3].

There are two mechanisms of cardiac involvement. Tumor extension through the renal vein and IVC is the main mechanism for the spread of cardiac ccRCC. On the other hand, metastases may also arise by diffuse systemic blood-borne spread or through the intrathoracic lymphatic system, especially in the presence of disseminated disease and pulmonary metastases [3]. It is clear that left ventricular metastases from ccRCC without vena caval or right atrial involvement are casuistic (Table 1). In the absence of either direct vena caval extension or systemic disease, involvement of the heart is extremely rare with only two cases known [12]. This specific pattern of metastasis should be considered a Stage 4 disease, with an expected five-year survival of less than 10% [20]. Up until now, only 21 cases of intramyocardial tumors of ccRCC have been described in the literature (Table 1). Majority of these cases were single tumors. Only reports by two groups have shown multiple cardiac metastases from ccRCC [3,13]. Moreover only in two cases, the patients were treated with TKI, but in both cases, cardiac tumors were single, and no cardiac tests were reported nor was functional testing evaluation performed. In one of the cases the tumor actually developed on sorafenib treatment [14]. Other disease course and treatment is required if is a tumor thrombus that protrude to the heart, but not develop as intramyocarial metastases. In particular it was shown that tumor thrombus level does not predict recurrence or mortality in this group of ccRCC patients who present with IVC involvement in the renal vein (Group 1) or subdiaphragmatic IVC tumor thrombus (Group 2), in comparison to involvement of IVC above diaphragm or atrial extension (Group 3). Survival is determined by inherent aggressiveness of the cancer manifested by tumor size, grade and distant metastasis at presentation [28]. Pre-surgical treatment with sunitinib is able to ease surgery for ccRCC tumor thrombi and surgery after sunitinib treatment may be possible without additional morbidity. In this setting, two courses of pre-surgical therapy with sunitinib may be appropriate treatment [29].

Most ccRCC cardiac metastases remain asymptomatic, as in the case of this patient at the time of diagnosis. The most common cardiac presentation of renal cell carcinoma is hypertension, but other cardiac presentations include shortness of breath, cough, arrhythmias, chest pain, important hemodynamic impairment, and peripheral edema [21]. Tachyarrhythmia is also found often in this patients and may lead to syncope [21]. Coronary occlusion or compression from tumor masses can lead to myocardial infarction, heart failure, and death [12]. A typical clinical pattern of cardiac ccRCC disease progression is characterized by a worsening performance status, exacerbation of cardiac symptoms, including tamponade, arrhythmia, obstruction, or dilated cardiomyopathy, representing one of the terminal events as in the case of the reported patient [30]. Coronary occlusion or compression from tumor masses may also lead to myocardial infarction and death [12]. Pericardial involvement with effusion and cardiac tamponade is the most commonly recognized cause of hemodynamic compromise [24].

A variety of diagnostic imaging and hemodynamic techniques have been applied in the diagnostic process of all cases published, including echocardiography, CT, MRI, and even right heart catheterization and right ventriculography [24]. In our opinion cardiac MRI could be the diagnostic tool of choice in the assessment of patients with RCC when cardiac metastasis is suspected (Figure 2). Although there is overlap of the MRI characteristics of several cardiac masses, MRI is reliable tool to exclude lipomas, fibromas, and hemangiomas as well as thrombus or lipomatous hypertrophy. Also necrosis, extra-cardiac spread, and pericardial effusion may be identified with cardiac MRI [31]. Transthoracic echocardiography, transesophageal echocardiography, and PET-CT may also be used to diagnose cardiac metastases [20,30]. The capability of PET for absolute quantification in general and for blood flow quantification in particular is a substantial advantage. As we have shown in this case with SPECT, cardiac lesions visualized by functional imaging are correlated with anatomic data and provides sensitivity as well as the specificity of scintigraphic findings. Myocardium perfusion and coronary morphology can be evaluated with SPECT/CT systems. For assessing absolute flow and coronary flow reserve imaging with SPECT also appears to be reliable [32]. Targeted systemic therapy may be useful for this group of patients [11], but profound necrosis in the heart wall may evoke tamponade, therefore follow-up cardiac MRI scans are required. Resection or embolization of ccRCC cardiac metastasis is dependent on the location and relationship of the tumor to the important local structures [11,19]. Finally, based on experience with this patient and literature data, we propose regular cardiac magnetic resonance imaging (MRI) evaluation in ccRCC patients receiving TKI treatment when intramyocardial ccRCC metastases are found. We believe that MRI combines noninvasive multiplanar imaging and the ability to acquire functional information with excellent contrast resolution [31].

Finally this case presents prolonged treatment with multiple lines of TKIs and mTOR inhibitor after immunotherapy. At this point of time third line therapy - following first-line TKI and mTOR inhibitors remains undefined, although sunitinib and other VEGF inhibitors have demonstrated activity in this setting [33]. Re-challenge with TKIs may provide clinical benefit in terms of PSF/OS after everolimus in patients with mRCC [34], as in this case. Recent data analysis has proven that globally PFS durations is shorter and response rate lower on re-challenge following initial treatment, but longer interval between treatments was shown to increase response to sunitinib re-challenge [35]. In a wide retrospective analysis in the sorafenib – mTOR inhibitor – sunitinib group subsequent PFS was of 11.7, 5.1 and 9.1 months, respectively, while in the sunitinib – mTOR inhibitor – sorafenib group PFS was 14.4, 4.3, and 3.9 months, respectively. This has led to conclusion that there is no significant difference between the two sequence modalities [36]. After failure of everolimus, re-exposure to TKIs was recently reported as a common clinical practice. It was demonstrates that patients obtain clinical benefit of therapies beyond immunotherapy – sunitinib/sorafenib – everolimus sequence. Drugs administered beyond was sunitinib (in 28.6% cases), sorafenib (in 28.6%) and other therapies (in 42.8%) in a recent report [37]. It was also shown that sunitinib and sorafenib re-challenge may be considered as it has had potential benefits in terms of PFS and may be tolerated in select mRCC patients [38,39]. This treatment modality may be effective due to a transient nature of sunitinib resistance [40,41] and in primarily sunitinib-responsive patients, re-challenge with sunitinib may be successfully introduced after mTOR inhibitor - refractory disease [42]. This case also confirms that sequential therapy enables to obtain prolong combined PSF as result of multiple lines of treatment. Although this patient obtained benefit from presented sequence immunotherapy – sunitinib – everolimus – sorafenib – pazopanib – axitinib, but for further cases ‘ideal sequence’ is still unknown. It should be hoped that TKIs, mTORIs with novel therapies (anti-PDL1, anti-PD1 anti-CTLA4) used in combination or sequentially have potential to provide best treatment and favorable outcomes in ccRCC. Results from ongoing and planned trials are expected to help shape future therapy [43].

## Consent

The patient gave consent for all treatment procedures used and future scientific publications. Informed consent for this publication was obtained from relative of the patient. The patient passed prior to manuscript preparation.

## Competing interests

CS has received honoraria for lectures from Pfizer, Roche, GSK, Novartis and Astellas. AMC has received honoraria for lectures from Pfizer, GSK, and Novartis. PS and FL indicate no potential conflict of interest.

## Authors’ contributions

Treatment: AMC, CS; Manuscript writing: All authors; Final approval of manuscript: All authors. Conception and design: CS, AMC; Collection and assembly of data: PS, AMC; Data interpretation: AMC, CS; Administrative support: PS, FL.

## Authors’ information

CS is a clinical oncology specialist since 1986, and has worked at Temple University School of Medicine, Jefferson Cancer Institute at Thomas Jefferson University, and in the last 10 years has participated in major renal cancer treatment clinical trials including AXIS, EU-ARCCS or TARGET and is an expert in renal cancer treatment; AMC specializes in clinical oncology and biotechnology, and has trained at the Universite degli Studi di Palermo, Paracelsus Medizinische Privatuniversität, and Emory University School of Medicine.

## Pre-publication history

The pre-publication history for this paper can be accessed here:

http://www.biomedcentral.com/1471-2490/14/73/prepub
